# Annotations

**Published:** 1905-08-05

**Authors:** 


### ANNOTATIONS.

### No Medical Women Need Apply.

An unusual incident happened at a meeting of the Chorlton Guardians, which was held last week, to consider the appointment of a junior medical officer at the Withington Workhouse. Five applications were sent in for the position, three from women, and two from men. But, before the qualifications of the candidates could be discussed, it was moved that only the two men should be sent for. A lady Guardian having moved, and another lady having seconded, that all the applicants should be asked to attend at the offices, Dr. Rhodes declared that he should object to having a woman doctor. This remark elicited from one of the lady Guardians a somewhat stinging rejoinder. The protest to which she took exception was, however, successful, the Guardians deciding by a small majority to send for the two male applicants only. In arriving at this conclusion, we think that the Guardians were wise, for it has been shown over and over again that, with existing domestic arrangements, the work of a medical institution is not, as a rule, performed with the necessary concord and efficiency if members of both sexes are included in the resident staff. The arguments against a mixed staff are sufficiently apparent to those who have had practical experience of one, and, while the objections are commonly attributed to prejudice or jealousy on the part of the male sex, it is certain that these motives do not usually enter into the question at all. Nevertheless, we admit that on the present occasion the fair sex have ground for complaint against the Chorlton Guardians. Their advertisement should have contained an intimation that no medical women need apply. This would have saved both the ladies who applied—not knowing that the barrier of sex would be raised—and the Guardians trouble, and we commend the precaution to other public bodies. Considerable trouble, expense, and loss of time are frequently caused to candidates, and especially to women, for medical appointments through ambiguity and careless wording of the advertisements. It is a sound principle that unless an appointment is not open to all qualified competitors the fact should be set forth when applications from persons desiring to fill it are invited.

### Standardisation of Medicines.

The main object and motive of the art of pharmacy may be fairly stated as the manufacture of remedies as prescribed by the physician in such a fashion that constancy of therapeutical activity shall be secured. Such an aim is readily and certainly obtained in the case of medicinal agents which have a definite chemical composition, and one that can be confidently estimated by ordinary analytical methods. But a different position is presented by many of the crude drugs from which such galenical preparations as tinctures, infusions, and extracts are commonly obtained. In some of these the active medicinal agent is either not known, or its quantitative appreciation is not possible, or, again, whilst the alkaloidal strength of the drug can be determined, there is reason to believe that this does not fully represent the total medicinal value of the remedy. Hence it arises that the therapeutic value of many galenical preparations is a varying quantity, even though they have been skilfully and carefully manufactured. The crude drug—say, for example, digitalis or belladonna—varies according to a number of circumstances, such as the soil in which it grows, the season in which it is collected, and so on. Necessarily, therefore, the preparations obtained by different pharmacists and at different dates are very likely to vary in medicinal efficacy. A position so serious demands most careful study, and it is satisfactory to learn that it is receiving the attention of a number of leading pharmacists. The desire is to obtain tests that will determine standards by which uniformity of medicinal strength can be secured. There are pharmacologists who anticipate that the physician of the future will use neither crude drugs nor galenical preparations made from them, but will rely upon definite active principles such as alkaloids or similar substances. It may, however, be doubted whether these bodies in all cases represent the whole of the medicinal virtues of the drugs which yield them, and in any event the abandonment of traditional methods is hardly close at hand.

### Old Wall-papers.

A rather important question was lately raised before Mr. Francis, at the Lambeth Police Court, on the application of the Southwark Borough Council for an order requiring the trustee of a large estate at Walworth to strip the walls of a room which was about to be repapered, on the ground that the previous coverings had become verminous. Mr. Francis made an order for the work to be done within seven days, but he declined to lay down any hard-and-fast rule on the subject, and it is believed that his decision will be appealed against. In many poor houses it is possible to find rooms in which layer after layer of new paper has been pasted over the old to avoid the cost of stripping; and it is an experiment worth trying to place a few fragments of this compound paper covering in a tin box and to smell them. The moisture necessary for removal, and equally necessary, of course, for producing the adhesion of a fresh layer, develops a stench from the old paste and the accumulated filth which must be experienced before it can be realised; and there can be no doubt that the presence on a wall of more than one layer of paper is quite sufficient to constitute a nuisance in a sanitary sense. Whether the organisms attendant upon the decomposition of which the smell is evidence would be noxious to mankind if they escaped into the room we cannot say; but it is quite certain that their escape would always be easy in the conditions supposed; and, altogether apart from the refinements of bacteriology, it is by no means unsound practice to accept the sense of smell as a valuable rough indication of what should be permitted or prohibited. The "vermin" might, of course, constitute an additional reason for the order made; but, however this may be, it is certain that the stripping of old paper is a measure generally necessary in the interests of health and of comfort.

